# New steps for the international consolidation of the Brazilian Journal of Pulmonology

**DOI:** 10.1590/S1806-37132014000400001

**Published:** 2014

**Authors:** Carlos Roberto Ribeiro Carvalho, Bruno Guedes Baldi, Carlos Viana Poyares Jardim, Pedro Caruso, Rogério Souza

**Affiliations:** Brazilian Journal of Pulmonology; Executive Editors; Executive Editors; Executive Editors; Executive Editors

In 2002, the Brazilian Journal of Pulmonology (BJP) was accepted for indexing in the SciELO database. This achievement, which is the result of the determination and efforts of the then current and previous Editorial Boards, was the basis for the construction of the Journal's image and for the Journal's international exposure. Subsequently, in 2006, the BJP was accepted for indexing in PubMed, which significantly increased the Journal's visibility. In 2012, after its inclusion in the Institute for Scientific Information (ISI) Web of Knowledge database, the BJP received its first impact factor, which placed the Journal in a very prominent position among the Brazilian scientific journals. This was extremely important, especially for respiratory researchers, because it meant the existence of a vehicle, with an international circulation, for communicating research results.

However, in 2013, because of questionable criteria, Thomson Reuters, which is the international company responsible for determining journal impact factors, did not publish the impact factor of the BJP.^(^ 1 ^,^ 2 ^)^ Although the Journal remained in the database, and therefore its publications and the citations of these publications were computed, the company chose not to publish the Journal's impact factor for that year. Considering that journal impact factors are used for journal classification by national funding agencies and for the evaluation of graduate programs, the consequences of such a penalty were huge, making it difficult to assess its extent. Unfortunately, these effects will still be felt in the coming years, unequivocally affecting subsequent impact factors.

Recently, on July 29, the annual Journal Citation Reports was published. The impact factor of the BJP for the 2013 publications was 1.268, which means that the BJP ranks sixth among the 107 Brazilian journals included in the ISI database. The return of the BJP to the annual list of the database that calculates impact factors shows, more clearly than do the ranking achieved and the impact factor itself, that the path followed by the Journal over the last few years has always been based on the dissemination of quality respiratory science. As previously mentioned, the impact factor of the BJP will still reflect the effects of that penalty over at least the next two years, because of the mechanism through which impact factors are calculated. Nevertheless, the return of the BJP to that annual list consolidates its position among the journals that are of greatest importance in disseminating knowledge in respiratory medicine worldwide.

In addition, in late July, SCImago cites per document index (which uses the Scopus database and is calculated in a similar way to the ISI impact factor) was published, and the BJP was assigned 1.45, reinforcing the Journal's position as one of the most important science journals in Brazil. This strengthens our responsibility with regard to the sustained growth of the BJP in the coming years.

To ensure this growth, structural changes have been made. We are in the final stages of changing the article submission system. As from September, we will use ScholarOne. This is the new platform that will be available to our authors, reviewers, and editors. The ScholarOne system is more modern and efficient, as well as being the model used by various journals of high international impact. The main goal of this migration is to facilitate and expedite the process of submission and review of articles, which is essential for extending the Journal's presence in the international arena, as well as being an added incentive for the participation of international authors and reviewers.

Another important achievement, which was completed on July 2014, is that the BJP content is now available through PubMed Central (PMC), which is the U.S. National Institutes of Health/National Library of Medicine (NIH/NLM) digital archive of biomedical and life sciences journal literature. This tool allows free access to full-text articles from the BJP, which surely increases the visibility of articles published by researchers from other countries, contributing to their dissemination. This is only possible because all articles published in the Journal are available in English.

The major responsibilities at this point, especially for the future Editorial Board of the BJP, are to consolidate our presence in international databases, such as SciELO, PubMed, Thomson Reuters ISI Web of Knowledge, Journal Citation Reports, and SCImago, and to continuously improve the quality of the published articles. To achieve these goals, the continuous participation of all researchers, reviewers, and editors who have contributed to the growth of the BJP throughout its history is essential. We hope we can count on the collaboration of more researchers so that the BJP can not only continue to be an important journal for the dissemination of respiratory research in Brazil, but also consolidate and increase its international exposure.
