# Supplementary material for: New steps for the international consolidation of the Brazilian Journal of Pulmonology
Source: J Bras Pneumol. 2014 Jul-Aug;40(4):325–6. doi: 10.1590/S1806-37132014000400001 (PMC4201164; doi:10.1590/S1806-37132014000400001)
Supplement: Translation

# Translation: Novos passos para a consolidação internacional do Jornal Brasileiro de Pneumologia

**Authors:** Carlos Roberto Ribeiro Carvalho, Bruno Guedes Baldi, Carlos Viana Poyares Jardim, Pedro Caruso, Rogério Souza

**Affiliations:** Jornal Brasileiro de Pneumologia; Jornal Brasileiro de Pneumologia; Jornal Brasileiro de Pneumologia; Jornal Brasileiro de Pneumologia; Jornal Brasileiro de Pneumologia

Em 2002, o Jornal Brasileiro de Pneumologia (JBP) foi indexado na base de dados SciELO. Tal fato, fruto da determinação e esforço do Corpo Editorial da época e dos anteriores, foi a base para a construção da imagem e a inserção internacional de nosso jornal. A partir daquele passo, em 2006, foi obtida a indexação no PubMed, promovendo uma expansão significativa na visibilidade do JBP. Em 2012, após a inclusão do JBP na base de dados do *Institute for Scientific Information *(ISI) *Web of Knowledge*, houve a liberação do seu primeiro Fator de Impacto, dando-nos uma posição de bastante destaque entre os periódicos científicos brasileiros. Esse fato foi de extrema importância, especialmente para os pesquisadores da área respiratória, pois indicava a presença de um veículo de circulação internacional para expor o resultado das pesquisas. 

Entretanto, em 2013, por critérios discutíveis, o Fator de Impacto do JBP não foi liberado pela Thomson Reuters, empresa internacional que é a detentora desse indicador.^(^ 1 ^,^ 2 ^)^ Apesar de nosso periódico ter permanecido na base de dados, sendo portanto computadas suas publicações e citações, houve a opção pela omissão do índice no referido ano. Considerando-se que o Fator de Impacto de uma revista é o índice utilizado para a classificação da mesma junto a agências de fomento nacionais e mesmo para a avaliação de programas de pós-graduação, as consequências de tal penalidade foram enormes, tornando difícil a avaliação de sua extensão. Infelizmente, essas repercussões serão ainda percebidas ao longo dos próximos anos, influenciando de forma inequívoca os índices subsequentes.

Recentemente, em 29 de julho, foi liberada a lista anual dos periódicos incluídos no* Journal Citation Reports*; fomos informados que a avaliação do JBP, relativa às publicações de 2013, apresentava um Fator de Impacto de 1,268, o sexto maior do Brasil entre os 107 periódicos nacionais incluídos na base de dados ISI. Mais que a posição obtida e o valor do indicador, a volta do JBP ao relatório anual da base de dados geradora do Fator de Impacto demonstra que o caminho trilhado ao longo dos últimos anos sempre foi pautado pela divulgação da ciência de qualidade na área respiratória. Conforme mencionado, o Fator de Impacto do JBP, pelo mecanismo através do qual é feito seu cálculo, ainda espelhará o efeito da penalidade sofrida ao longo de pelo menos os próximos dois anos; ainda assim, o retorno do JBP consolida sua posição dentre as revistas de relevância para a divulgação da área de medicina respiratória no mundo. 

Também foi liberado, nas últimas semanas do mês de julho, o índice "cites/document" da base de dados Scopus (que utiliza o mesmo método de cálculo do Fator de Impacto), liberado pela SCImago, e esse foi 1,45, reforçando a posição do JBP como uma das mais importantes revistas brasileiras de divulgação científica, fato que reforça nossa responsabilidade com o crescimento sustentado do JBP ao longo dos próximos anos.

Para garantir tal crescimento, mudanças estruturais foram realizadas. Estamos na fase final de mudança do sistema de submissão de artigos. A partir de setembro, utilizaremos o *ScholarOne*. Essa será a nova plataforma que estará disponível para nossos autores, revisores e editores. O sistema *ScholarOne* é mais moderno e eficiente, e é o modelo utilizado por diversos periódicos de grande repercussão internacional. O principal objetivo dessa migração é facilitar e agilizar o processo de submissão e revisão dos artigos, o que é fundamental para ampliar a presença do JBP no cenário internacional, inclusive funcionando como um maior atrativo para a participação de autores e revisores internacionais. 

Outro fator de grande relevância que foi concluído em julho de 2014 foi a disponibilização do JBP no PubMed Central(r) (PMC), que é o arquivo livre de periódicos de ciências biomédicas e da vida do *National Institutes of Health/National Library of Medicine *(NIH/NLM, Instituto Nacional de Saúde da Biblioteca Nacional de Medicina) dos EUA. A utilização dessa ferramenta permite o acesso livre aos artigos completos do JBP, o que seguramente aumenta a visibilidade dos artigos publicados por pesquisadores de outros países, contribuindo para sua divulgação. Isso só é possível porque nossos artigos são disponibilizados em língua inglesa.

As grandes responsabilidades que se impõem nesse momento, especialmente para o futuro corpo editorial do JBP, são consolidar nossa presença nas bases de dados internacionais, tais como SciELO, PubMed, Thomson Reuters *ISI Web of Knowledge, Journal Citation Reports* e SCImago, e a busca contínua pela maior qualificação dos artigos publicados. Para atingir esses objetivos será fundamental a contínua participação de todos os pesquisadores, revisores e editores que contribuíram ao longo de toda a história do JBP para seu crescimento. Esperamos contar com a colaboração de novos pesquisadores para que possamos não apenas nos manter como um periódico importante para a divulgação da pesquisa brasileira na área respiratória, como também efetivarmos e ampliarmos nossa inserção internacional.
